# Supplementary material for: Are carbon emissions trading and green financial instruments synergistic? -Comprehensive quantitative research based on content analysis
Source: PLoS One. 2024 Mar 7;19(3):e0298601. doi: 10.1371/journal.pone.0298601 (PMC10919601; doi:10.1371/journal.pone.0298601)
Supplement: S1 Data — (ZIP) [file pone.0298601.s001.zip › Data/地级市Z_F工作报告.zip!/╔╜╢1⁄2/╠⌐░▓╩╨/└┤╫╘╣1⁄2╓┌║┼í╛┬φ┐╦╩2╛▌═°í┐_2014.docx]

各位代表：

现在，我代表市人民政府向大会报告工作，请予审议，并请各位政协委员和其他列席会议的同志提出意见。

一、2013年工作回顾

2013年，面对复杂的形势，在市委坚强领导下，全市上下紧紧围绕推进富民强市、建设幸福泰安奋斗目标，开拓创新，务实苦干，全市经济社会各项事业协调推进。

经济运行稳中有进。预计全市实现生产总值2820亿元，增长10.6%左右；完成公共财政预算收入168.8亿元，其中税收完成118.4亿元，占公共财政预算收入的70.16%，同比提高4.84个百分点；完成固定资产投资2030亿元，增长20.7%左右；实现社会消费品零售总额1050亿元，增长13.1%左右；城镇居民人均可支配收入28225元，增长10%左右；农村居民人均纯收入11500元，增长13%左右；人民币各项存款余额2261亿元、贷款余额1416亿元，分别比年初增加327亿元和189亿元。

农业生产得到巩固提高。粮食总产达到308.7万吨，实现“十一连丰”，岱岳区、肥城市被评为全国粮食生产先进县。全市有机蔬菜基地面积达到35万亩，苗木花卉35.4万亩，茶园2.3万亩，奶牛存栏25.8万头。规范流转土地承包经营权面积91.2万亩，家庭农场达到953家，农民合作社达到4852家，规模以上农业龙头企业达到712家。新发展“旱能浇、涝能排”高标准农田20万亩、高效节水灌溉面积21万亩，治理水土流失面积80平方公里，解决了20.2万农村人口饮水安全问题。大汶河综合开发已建成唐庄等6座河道拦蓄工程，形成了长50公里、静态蓄水8000万方的大水面。新增造林面积20.9万亩，其中经济林面积7万亩，全市森林覆盖率达到38.9%。改造农村公路580.8公里。强农惠农富农政策全面落实。

工业经济不断升级壮大。预计规模以上工业企业实现主营业务收入6260亿元、利税705亿元、利润444亿元，分别增长13%、5%和6%左右。新增规模以上工业企业250家左右；新增主营业务收入过10亿元、利税过亿元的企业16家和13家；泰开集团、肥矿集团、瑞星集团主营业务收入突破百亿元，百亿企业达到5家。输变电设备、汽车、非金属新材料等传统优势产业加快技术改造，全年实施工业在建技改项目991项，预计完成投资660亿元。装备制造业效益大幅增长，对规模以上主营业务收入增长的贡献率达到40.9%。信息产业发展迅速，泰安云计算中心一期投入运营，泰盈科技成为国内规模较大的呼叫服务中心，泰山云谷互联网产业园开工建设。新能源产业发展势头较好，泰安高新区列为国家首批分布式光伏发电示范区，东平大唐风电顺利并网运行。科技创新能力不断增强，国家级高新技术企业达到95家，省级以上创新平台达到73家，中国驰名商标达到26件；新增发明专利授权量260件，国家知识产权示范创建市通过验收；山东蓝光软件获国家科技进步二等奖，康平纳集团成为我市首家国际科技合作基地；国家再制造机械产品质量监督检验中心、国家级高创中心孵化基地、中国航天特车研究院、山东省矿用提升输送设备监督检验中心等一批研发检验机构正在建设。狠抓工程减排、管理减排和结构减排，完成了省下达的节能减排目标任务。

服务业发展有新提升。旅游业稳步发展，太阳部落、宝泰隆旅游度假区、刘老根大舞台建成运营；整合旅游资源，推出多种“中华泰山、天下泰安”三日游产品，增强了旅游项目集约效应；预计全市接待境内外游客4836万人次，实现旅游总收入441亿元，分别增长11%和14%。启动创建首个国家级旅游服务综合标准化示范市，旅游满意度持续提升，泰山被评为全国旅游服务质量标杆单位。现代物流业发展迅速，全市在建商贸物流园区24个，计划总投资超过370亿元，岳华能源物流、杭州长城五金机电商城、中富宏泰建材城等基本建成；泰安港被交通运输部和省政府确定为地区性重要港口。文化产业加快发展，泰安传媒集团成立，泰山文化产业园、泰山区科技文化产业园、泰安高新区国家级文化创意产业园正在建设。

招商引资和项目建设取得新成效。预计全市121个市级重点项目完成投资460亿元，100个前期推进项目中80个已开工建设。中石油天然气LNG、国电热电联产、沃尔智慧停车系统、泰山玻纤产业园、中国国际时尚产业设计创意园、山能机械装配再制造、恒基新材料、王老吉系列饮品、光大集团大豆制油精加工、奥特莱斯现代服务产业区等项目建设顺利。加大产业招商力度，举办和参加了中国泰安投资合作洽谈会、（北京）泰安经济合作洽谈会、（上海）泰安经济合作洽谈会、香港山东周、台湾山东周等招商活动，签约投资亿元以上项目111个。

改革开放取得新成绩。加大中小微企业扶持力度，民营企业加快发展，全市新登记私营企业3691家，主营业务收入过50亿元的产业集群达到18个。开展了“营改增”试点，共有4216户企业纳入试点范围，减税1154万元。深化政府投融资改革，加快融资平台转型，组建融资租赁公司，支持了实体经济发展。全市20个乡镇、40个村开展了农村土地承包经营权股份制改革试点，80个乡镇、417个村进行了农村土地承包经营权颁证工作。实施了集体林权配套改革，6个县市区都成立了林权管理服务中心或林权流转平台，发展林业专业合作经济组织856个，参加农户12.6万户；13处国有林场列入国家级改革试点。医改工作稳步推进，全市88家政府办基层医疗机构和2806个实行一体化管理的村卫生室全部实行基本药物制度；全面完成了公立医院编制核定工作，已有5家县级公立医院进行了改革试点。开放型经济实现逆势突破，预计完成合同利用外资4.9亿美元，实际到账3亿美元，分别增长118.7%和76.7%左右；完成进出口总值25亿美元，增长15%左右；完成境外投资1.06亿美元，增长100%。成功举办了首届中国（泰山）国际矿业装备与技术展览会，国内外1000多家企业参展。我市跻身中国外贸竞争力百强城市。积极融入省会城市群经济圈和省西部经济隆起带，扎实推进“一圈一带”改革开放先行区建设。泰安高新区坚持高端发展、特色发展，泰邦生物科技园、日本生态新材料工业园、中国五矿甬泰新材料科技园等一批高新技术项目落地建设。

城乡建设呈现新面貌。强化泰城中心带动作用，编制完成城市空间发展战略研究和汶河新区、104国道沿线新区等片区规划。时代发展线万达广场、中南集团财源街中央商务区、泰安高新区中国五矿城市综合体、青春创业园天房集团城市综合体落地建设。新华片区、恒大片区、宝盛广场、高铁片区等10大片区建设顺利。泰城道路工程加快建设，樱桃园路、环山路东延、泰新路泰城出口段全线贯通，龙潭路地下停车场主体完成。泰城引水工程主体完工，三合水厂改造工程投入使用。泰城配电网自动化实现全覆盖。积极开展国家卫生城、国家环保模范城市、国家森林城市、国家生态园林城市、现代水利示范市、水生态文明城市创建，城乡环境明显改善，泰安被授予中国人居环境奖。各县市区加大城市基础设施投入，县城驻地和乡镇建设呈现新面貌。新型农村社区建设取得新进展，全市累计开工建设新型农村社区402个，其中已建成集中居住式农村社区222个。全面启动生态泰安建设，加大水污染和大气污染防治力度，扎实开展生态示范建设，建成国家级、省级生态乡镇59个。

民生保障和社会建设得到加强。全市财政用于重点民生事项支出达到143.5亿元，占公共财政支出的55.4%。为民要办的10件实事基本完成。预计新增城镇就业6.3万人，农村劳动力转移就业9.2万人。各项社会保险扩面14.2万人次，发放各项社会保险金82亿元；在全省率先建立城镇居民大病医疗保险制度，推行了城镇基本医疗保险门诊统筹，扎实做好了居民社会养老保险工作；城乡低保、五保、孤儿、残疾人等保障水平进一步提高；社会养老服务体系建设全面启动，新增养老床位5560张，山东泰山社会福利中心开工建设。全面完成学前教育三年行动计划，新建和改扩建公办幼儿园484处；加快推进学校标准化建设，肥城市、宁阳县通过国家义务教育均衡发展评估验收；实施职业教育学校实训设备配备工程，全面启动了中高职教育衔接。基层卫生公共服务体系不断完善，创建全国示范社区卫生服务中心2个，我市荣获全国基层中医药工作先进单位，新泰市被授予国家卫生应急综合示范市称号；新农合筹资标准达到每人350元，基本公共卫生服务经费标准提高到人均30元。基层公共文化服务体系建设进一步加强，新建改建文化场馆20个；泰山报业文化中心、泰安文化艺术中心建成运营，承办了第十届中国艺术节有关赛事，成功推出“泰山情缘之石敢当”音乐剧，被表彰为首批文化强省先进市。全市人口低生育水平连续23年保持稳定，连续13次获得全省人口目标责任考核先进单位称号。深入实施农村健身设施覆盖工程，被授予全国群众体育先进单位。开工建设保障房10121套，基本建成8361套，高铁片区保障房项目获国家建设工程质量最高奖“鲁班奖”；完成农房建设4.2万户、危房改造1.3万户；发放住房公积金贷款13.2亿元，“零余额账户”管理经验在全国推广。加强社会组织管理创新，全面推行了城乡社区网格化服务管理模式；实施“天网工程”，平安泰安建设成效显著。落实安全生产责任，加大隐患排查整治，杜绝了较大以上事故的发生，全市安全生产形势持续稳定。理顺管理体制，食品药品监管得到加强。双拥共建、民兵预备役、国防动员、人民防空、老龄、妇女儿童、新闻出版、民族宗教、外事侨务、气象、地震、档案、史志等工作取得新成绩。

政务服务环境进一步优化。深入开展“行政程序年”活动，政府依法行政能力不断提升。出台了《进一步提高行政审批服务效率优化发展环境的意见》，减少行政审批事项87项，取消行政事业性收费30项，行政服务效率明显提高。认真开展群众满意度调查、机关作风和政风行风评议，扎实开展“四风”问题和“慵懒散”专项治理，加大对政务督查、行政审批、便民服务、应急管理工作考核力度，政府服务和落实能力进一步增强。

各位代表，过去一年我们取得新的成绩，得益于市委的坚强领导，得益于全市人民的共同努力。在此，我代表市人民政府，向全市广大干部群众、各民主党派、工商联、无党派爱国人士，向人民解放军和武警驻泰部队及驻泰单位，向关心支持泰安发展的港澳台同胞、海外侨胞及海内外朋友，表示崇高敬意和衷心感谢！

在肯定成绩的同时，我们也清醒地看到，经济社会发展仍然存在一些不容忽视的问题：经济总体实力还不强，骨干企业较少、核心竞争力偏弱，发展中不平衡、不协调、 不可持续的矛盾仍然存在；经济增长的资源、环境及生产要素约束加大，节能减排形势严峻，企业创新活力不足，推动经济转型升级任务艰巨；体制性、机制性障碍依然存在，改革开放的力度需要进一步加大；社会管理和民生工作存在一些薄弱环节，公共服务体系还不够完善；政府职能转变还不到位，行政服务效能需要进一步提高。对这些问题，我们要强化机遇意识、责任意识和进取意识，坚持用改革的思路和发展的办法，认真加以解决。

二、2014年政府工作总体安排

今年是深入贯彻落实党的十八届三中全会精神、全面深化改革的第一年，也是完成“十二五”规划的关键一年。政府工作总体要求是：全面贯彻落实党的十八大、十八届三中全会和习近平总书记视察山东讲话精神，坚持以邓小平理论、“三个代表”重要思想、科学发展观为指导，紧紧围绕推进富民强市、建设幸福泰安奋斗目标，以提高发展质量和效益为中心，坚持稳中求进工作总基调，进一步解放思想，全面深化改革，坚决打好转方式调结构攻坚战，继续抓好招商引资和项目建设，做好民生改善和社会治理工作，着力激发市场活力，统筹城乡发展，扩大对外开放，加快生态文明建设，促进全市经济持续健康发展、社会和谐稳定。

2014年主要预期目标是：生产总值增长9.5%左右；公共财政预算收入增长11%左右；规模以上固定资产投资增长18%左右；社会消费品零售总额增长13%左右；实际利用外资增长20%左右，进出口总值增长11%左右；城镇居民人均可支配收入增长10%左右，农民人均纯收入增长11%左右；城镇新增就业5.2万人，新增农村劳动力转移就业6.5万人，城镇登记失业率控制在3.5%以内，人口自然增长率控制在10.03‰以内；全面完成省政府下达的年度节能减排和大气污染防治约束性目标任务。

2014年，世界经济仍将延续缓慢复苏态势，我国经济处于增长速度换挡期、结构调整阵痛期、前期刺激政策消化期叠加阶段，经济下行压力较大。实现今年发展目标，要按照习总书记“腾笼换鸟、凤凰涅槃”的总要求，以改革创新的思维，创造性推动各项工作。在工作指导上要实现五个新转变：一是在发展理念上实现新转变，坚持以提高经济发展质量和效益为中心，形成持续提质增效的发展局面；二是在谋划改革上实现新转变，坚持正确准确、稳妥有序推进改革，把握好节奏和时机，通过改革解决制约泰安发展的重大问题，进一步释放发展活力；三是在发展动力上实现新转变，大力实施创新驱动发展战略，加快实现由“泰安制造”向“泰安创造”、“泰安品牌”转变，不断增强发展内生动力；四是在发展途径上实现新转变，扭转产业结构偏重、层次偏低的状况，着力推动三次产业结构从“二三一”向“三二一”转变，产品结构向价值链高端转变，管理方式向现代管理转变；五是在考核导向上实现新转变，改革完善科学发展综合考核体系，强化审计问责机制，重视看基础、看质量、看潜绩、看实绩，用好科学的政绩考核“指挥棒”，激励全市上下干事创业。

三、加快经济转型升级步伐

（一）做强做大工业，扭转工业经济薄弱局面。工业是全市经济的支柱，也是产业转型升级的主战场。要坚持不懈抓优质投入、上高效项目、降过剩产能，以优质增量带动结构优化和发展方式转变。一是完善工业发展规划。以各类园区为依托，突出比较优势，优化工业布局，最大限度防止市内各区域之间产业雷同、重复建设和资源配置浪费，打造一批配套程度高、规模优势突出、核心竞争力强的特色产业聚集区。二是增强产业发展优势。加大传统产业升级改造，推动技改由生产制造环节向价值链高端延伸。完善落实输变电设备、纺织服装、化工、食品等产业招商和项目建设规划，补齐产业短板，拉长产业链条，形成产业集群。把加快发展新材料、新能源与节能环保、电子信息、生物医药与生物育种等战略性新兴产业，作为工业转型升级的主攻方向，形成工业新的快速增长极。三是加大骨干企业培植力度。坚持“一企一策”，落实各项扶持措施，帮助企业解决发展难题，加快形成一批支撑带动力强的产业集群和大型企业集团。力争到2015年，主营业务收入过百亿元的企业达到8家，过50亿元、30亿元的企业户数明显增加。四是提高企业组织化程度。加强工业联盟和行业协会商会建设，条块结合构筑平台，丰富政府服务企业的手段，引导同区域、同行业企业加强交流、共享资源、抱团发展。鼓励支持企业既要重视生产和营销，更要注重研发和服务，促进企业现代化运营管理。

（二）大力推动服务业发展，增强消费对经济的拉动作用。一是努力提升旅游业。围绕建设国内外著名旅游目的地，实现旅游业发展由人数增长型向消费增长型转变，由景点分散发展向产品整合营销转变，由注重景区建设向构建旅游目的地体系转变。高标准编制《泰安市旅游业发展总体规划》，促进全市旅游业持续健康发展。加快泰山古镇、泰山龙曦国际旅游度假地、泰山千古情城、快乐驿站、虎山公园扩建、岳海新天街等旅游项目建设；深度规划打造碧霞湖旅游度假区、徂徕山温泉度假区、新泰莲花山旅游度假区、肥城春秋古城、宁阳神童山风景区、东平白佛山景区等景区景点。加快发展乡村旅游，打造“泰山人家”品牌。积极做好泰安三日游产品推介营销工作。深入实施旅游满意度提升工程，积极创建国家级旅游服务综合标准化示范市。完善特色美食街、旅游文化商品城、旅游公交设施、旅游标识导向系统等服务设施，发展泰山玉、泰山茶、泰山螭霖鱼产业。二是大力发展商贸物流业。编制全市商贸物流发展规划，抓好现代流通体系建设，培育壮大一批龙头物流企业和物流园区，重点抓好新加坡泺亨现代物流园、泰安高新区物流园、速恒现代物流园、麦德龙超市仓储物流园、润恒冷链物流产业园、新泰天宝煤炭物流园、肥城齐鲁国际农产品冷链物流园、泰安港物流园、快递物流园等项目建设。大力发展电子商务、连锁经营等现代交易方式，推动商品交易转型升级。积极培育泰安会展品牌，发展会展经济。研究解决制约我市物流业发展的环境政策问题，建设物流信息平台，降低物流成本，提高物流效益。编制城乡商业网点规划，建设城市特色商业街区、社区超市和乡村超市；继续实施万村千乡市场工程，规范农村市场，促进农村消费。三是着力培植文化产业。加强文化与农业、工业、旅游等产业融合，大力实施泰山文化走出去战略，支持发展具有浓郁地方特色和较强影响力的文化产业。深化文化体制改革，培育文化市场主体，引进一批知名文化品牌，发展一批文化创意骨干企业，建设一批文化产业园区。四是加快培育各类服务业。落实服务业发展扶持政策，探索服务业发展新模式，扎实开展泰山区省级服务业综合改革试点。加快发展教育、文化、医疗等公共服务业，提升发展商贸餐饮、家政服务、健康养生等生活性服务业，扶持发展金融、信息、创意等生产性服务业，积极培育商务、科技、慈善及社区服务等中介服务业。

（三）全面提升开放水平，实现开放型经济新突破。大力实施开放兴市战略，落实鼓励扶持政策，突出引外资、促外贸、扩外经、强园区，构筑以发展开放型经济为中心的现代开放体系。坚持请进来和走出去并重，创新利用外资方式，集中优势资源招大引强，力争在引进战略投资、世界500强和跨国知名企业等方面实现新的突破；鼓励支持企业到境外投资办厂、设立研发机构、扩大对外工程承包，培育本土跨国品牌企业；拓展国际劳务市场，提高劳务合作效益。坚持出口和进口并重，加快培育进出口龙头企业，扩大出口规模；大力发展加工贸易，优化出口产品结构，着力抓好机电出口基地和出口农产品质量安全示范区建设；鼓励支持先进技术设备、关键零部件和能源原材料进口。坚持对外开放和对内开放并重，加强与国内先进地市开展合作，与省西部经济隆起带和省会城市群经济圈各地一体融合、共赢发展；用好沂蒙革命老区、国家中原经济区、资源枯竭型城市、山东半岛蓝色经济区联动示范基地等政策，提升区域经济整体实力。完善园区配套服务设施，抓好国际经济合作园区和海关监管场站、综合保税区等规划建设。

（四）高质量抓好项目建设，积蓄经济持续发展后劲。进一步健全指挥部工作机制，完善领导包保重点项目制度，重点解决好项目建设手续办理、资金土地、施工环境中存在的问题，集中力量抓好120个左右市级重点项目、100个左右前期推进重大项目。强化项目联合审查制度，严格国家产业政策和我市产业发展规划，严格项目投资密度和效益标准，严格项目科技含量和环境评价。加大招商引资力度，认真研究产业调整升级新规律、国内外领军企业规划发展新动向，面向国内外知名大企业开展上门招商、驻点招商。围绕培植产业龙头企业、延伸产业链条和发展高端高质高效产业，深度做好产业招商规划和项目策划。进一步完善产业招商工作体制，充分发挥企业主体作用，引导和帮助企业利用国内外企业跨国并购、增资扩股、股权投资、资产重组、经营合作、上市融资等多种方式开展合资合作，争取再有一批招商大项目、好项目落户泰安。

（五）强化财税金融支撑，保障经济社会平稳发展。一是加大财源培植力度。坚持把壮大财源与转方式调结构相结合，稳定财源建设资金投入，加快政府投融资转型，更多地把财政扶持重点转到科技创新、节能减排、人才引进培养、公共服务和基础设施上来，引导优质资源向创税产业集聚。落实结构性减税政策，减轻企业和社会负担。二是坚定不移调整财税结构。按照提高“两个比重”、确保“两个高于”的要求，强化工商税收特别是主体税收征管，保持优质税收稳定增长，提高财政收入质量，力争全市公共财政收入中税收占比提高到72%以上。进一步优化财政支出结构，大力压减一般性支出，特别是“三公”经费支出，降低行政运行成本，集中财力保重点支出。加强政府性债务管理，完善规模控制和风险预警机制，防范债务风险。三是深化财税体制改革。扎实推进税制改革，重点做好“营改增”试点扩围、后续管理工作；深化预算管理改革，探索建立全过程预算绩效管理体系；深化收支两条线、国库集中支付、政府采购、财政投资评审、公务卡等改革，提高财政资金使用效益。以管资本为主，健全完善国有资产管理体制机制，加大国有企业改革力度，提高国有资产效益。四是大力支持金融业改革发展。加强融资服务创新，着力抓好项目融资和银企合作，确保大项目建设和企业生产资金需求。积极开展直接融资，争取力博科技、一滕新材料实现上市，培育一批“新三板”和股权挂牌企业。加快发展小额贷款公司、融资性担保公司、民间资本管理公司等新型地方金融组织。深化地方法人金融机构改革，做强做大市商业银行，全面推进5家县级农信社银行化改革。积极引进异地金融机构落户，拓展融资增长空间。抓好泰安高新区金融创新示范区和新泰市省金融创新示范县建设，规划建设泰安市金融中心。扩大农业政策性保险，拓宽保险服务领域。优化金融生态环境，建立健全地方金融监管体制，加强地方金融风险防范。

（六）鼓励支持创业创新，激发全社会创造活力。一是激励创业。改革工商登记制度，降低创业门槛，加强市场秩序监管，建立统一开放、竞争有序的市场体系。实施民营企业倍增计划，加大中小微企业创业辅导基地和创业园建设力度，在财政扶持、税收优惠、公共服务、企业融资和产业引导上更多地向中小微企业倾斜，推动全民创业。二是推动创新。深化科技体制改革，建立以企业为主体、市场为导向、产学研相结合具有泰安特色的区域创新体系。增加科技投入，建立主要由市场决定项目设立、经费分配和成果评价机制，鼓励企业加大研发投入，培育更多创新型企业。加快科技创新载体建设，支持企业建设省级以上工程技术研究中心、重点实验室等高水平研发机构，在全市重点产业领域认定和创建一批产业技术研究院，在各类园区建设一批科技孵化器和公共服务平台。培育发展国家级高新技术企业，支持企业承担国家、省级重大科技项目。加强知识产权保护运用，创建国家知识产权示范城市。支持驻泰高校发展，为科技成果就地转化创造条件。加强创新人才队伍建设，重点引进“千人计划”、“泰山学者”等海内外高端人才，积极引进一批企业急需的应用型人才。完善企业家培养、交流、考核和奖励机制，建设一支有远见、善创新、懂经营、会管理的战略性企业家队伍。

四、稳妥推进城乡统筹发展

（一）着力提高城镇化发展质量。强化泰城中心城市辐射带动作用，坚持片区开发、组团建设，突出规划的引导调控作用，优化城市空间和功能布局，推动泰城由“依山而建”向“依山傍水”发展。历史文化轴重在体现城市文脉，提升文化品位；时代发展线重在加快开发建设，打造城市核心区；东西两翼重在利用交通区位优势，加快拓展开发；泰安高新区新城区重在完善设施配套，产城一体发展；汶河新区重在突出山水生态优势，建设休闲度假地。加快推进万达、五矿、中南、天房四大城市综合体和重点片区开发，注重抓好片区统筹规划和综合配套。继续加强城市道路建设，实施京沪高铁泰安站改造、万官大街、灵山大街东段、花园路等工程。继续实施引水进城和汶河综合开发工程，打造城市水景观和水文化。加快泰城应急水源地、供水排水、供热管网、环卫设施等建设，完成第一、二污水处理厂升级改造，开工建设第四污水处理厂。完善主要由市场决定价格机制，建立健全居民用水、用电、用气阶梯式价格制度。加大城市环境综合整治力度，加快城区农贸市场建设。优化调整公交线网布局，推进公交线路向居民小区、旅游景区（点）、学校及周边乡镇延伸。

创新城镇建设管理模式，吸引更多社会资金参与新型城镇化建设。抓好新型农村社区建设，配套完善基础设施和公共服务设施，同步发展特色产业园区，引导农村居住向城镇集中、向农村社区集中、向中心村集中。改革户籍制度，有序推进农业转移人口市民化，逐步实现教育、医疗、社保、邮政便民服务等基本公共服务覆盖全部城镇常住人口。加强生态文明乡村建设，打造一批特色美丽乡村。

（二）抓好农业农村改革发展。坚持“三个导向”，始终把解决好“三农”问题作为工作重中之重。以保障粮食安全和促进农民增收为核心，积极开展粮食高产创建活动，落实粮食最低保护价政策。完善特色农产品发展布局规划，加快农业结构调整，年内新认证有机食品1万亩，新建茶园0.5万亩。健全农产品质量标准、检验和认证三大体系，整体建设农产品质量安全区。加强重大动物疫病防控，确保畜牧业生产安全。实施项目带动工程，引进培植一批农业龙头企业，抓好新希望集团100万头生猪养殖基地、滕头和东方园林苗木基地、益客畜禽产品加工产业新城等项目建设，新建扩建规模以上农业龙头企业60家，建设一批农业特色园区。完善基层农技推广体系和运行机制，提高农业机械化水平，加大新型职业农民培训力度。做好农村土地承包经营权确权登记颁证工作，鼓励土地承包经营权向新型经营主体流转，年内新发展农民合作社400家，新注册家庭农场100家；做好农村集体土地所有权、宅基地使用权和建设用地使用权确权登记颁证工作，建设农村产权交易市场，着力抓好东平县农村综合产权交易所运营管理。深化征地制度改革，提高农民在增值收益中的比例。深化供销社改革，以土地托管、领办合作社为切入点，促进农业服务规模化。统筹抓好中小河流治理和水库除险加固等工程，建设“旱能浇、涝能排”高标准农田20万亩，治理水土流失面积80平方公里，解决10万农村人口饮水安全问题。深入开展国家森林城市创建，完成成片造林12万亩。积极实施村级公路网化示范县工程，新建改建农村公路695公里。切实抓好强农惠农富农政策落实，维护农民合法权益。

（三）加强生态文明建设。坚持绿色发展、低碳发展，开展循环经济示范建设活动，加快建成一批循环经济园区、清洁生产示范园区。坚决淘汰落后产能，严禁新上高耗能、高污染项目。积极推动节能技术进步，突出抓好工业、建筑、交通、公共机构等重点领域和重点企业节能。大力发展光伏、风电、生物质能等清洁能源。搞好人工湿地建设，加快建成柴汶河、天泽湖、康王河等人工湿地水质净化工程。实施大气污染防治“蓝天工程”三年行动计划，落实重污染天气应急预案，抓好重点行业、重点区域大气污染尤其是扬尘污染防治。加大生态泰安建设力度，健全生态文明制度，完善生态补偿机制，加快生态示范创建，新增省级以上生态乡镇6个、市级以上生态村30个。巩固提升国家环保模范城市创建工作，争取通过国家验收。实施最严格水资源管理制度，切实抓好水资源利用保护。强化项目用地效益审查，探索更加有效节约集约利用土地的制度和办法。

五、着力抓好以民生为重点的社会建设

（一）努力办好幸福民生。巩固创业型城市创建成果，健全政府促进就业责任制度，加强大学生创业孵化基地、返乡农民工创业园等载体建设，完善就业创业优惠政策和公共服务体系，加大就业创业能力培训力度，统筹做好高校毕业生、农村转移劳动力、城镇困难人员、退役军人就业。完善居民基本养老保险制度，整合城乡居民基本医疗保险，健全大病医疗保险制度，规范各项基本社会保险统筹层次，建立社会保障待遇确定和正常调整机制；稳妥推进收入分配制度改革，健全完善最低工资制度、企业工资集体协商制度、机关事业单位津贴补贴制度。抓好幸福教育，加快义务教育学校校舍标准化和信息化建设，扩大优质教育资源覆盖面，建设泰安一中新城校区；深化教育教学改革，全面实施素质教育，促进学生全面发展；大力发展职业教育，建设特色职业教育名校；鼓励社会力量多种形式兴办教育。深入推进县级公立医院改革，巩固基层医疗机构实施基本药物制度成果；大力扶持发展中医药事业；完善突发公共卫生事件应急和重点传染病防控机制。进一步提高城乡低保标准和补助水平，健全城乡特殊困难群体临时救助制度。继续做好0-6岁贫困残疾儿童抢救性康复救助工作，抓好市县残疾人康复中心、托养中心和市特教中心建设。鼓励引导社会力量参与养老服务，加快建立以居家为基础、社区为依托、机构为支撑的社会养老服务体系；着力抓好山东泰山社会福利中心、城市社区老年人日间照料中心和农村幸福院建设，新增养老床位4000张，新建农村幸福院500个。实行公租房和廉租房并轨，完成省下达的保障性住房建设任务；加快农村住房建设和危房改造；扩大住房公积金制度覆盖面，加强资金使用管理。

（二）创新社会治理方式。加强城乡社区规范化、网格化管理，完善社区公共服务体系。深化社会组织管理创新，支持和发展志愿者组织，大力推广“菜单式”志愿服务工作；重点培育和优先发展行业协会商会类、科技类、公益慈善类、城乡社区服务类社会组织，推动政府购买社会组织服务。创新信访工作制度，积极探索多元化排查调处工作机制，有效预防和化解社会矛盾。深入推进平安泰安建设，加强社会治安综合治理，强化应急管理工作。完善食品药品监管体制，建立最严格食品药品安全监管制度。健全安全生产监管机制，深化重点领域隐患排查和专项整治，确保安全生产形势持续稳定。

（三）繁荣发展其他社会事业。大力实施文化惠民工程，创新完善公共文化服务体系，加快基层文化设施建设，扎实开展公共文化服务提升年活动。坚持计划生育基本国策，稳定低生育水平，深入开展“和谐人口、幸福计生”创建工作。加强体育基础设施建设，推动竞技体育、群众体育和体育产业协同发展。认真抓好国防动员、人民防空、民兵预备役、双拥共建和优抚安置工作。促进民族团结进步，依法管理宗教事务。关心支持老龄、妇女儿童、残疾人事业发展。抓好全国第三次经济普查。做好外事侨务、爱国卫生、防灾救灾减灾等各项工作。

六、努力加强政府自身建设

形势的发展、事业的开拓、人民的期待，都要求我们以改革创新的精神全面加强政府自身建设，进一步提高政府施政能力和水平。

一要切实转变政府职能。更加注重发挥市场配置资源的决定性作用，该由市场做的放给市场，该由企业做主体的政府不能过多干预，社会能办好的交给社会，重点抓好事业单位分类改革和行业协会、中介组织与行政事业单位脱钩工作。要进一步深化行政审批制度改革，加强各级政务服务平台建设，完善重点项目和外来投资项目绿色通道制度，努力打造高效优质、重诺守信的营商环境。要切实加强公共资源交易统一管理，规范中介市场秩序，进一步激发市场主体发展活力和创造能力。

二要切实提升施政理念。树立正确的政绩观和发展导向，纠正唯GDP、唯数字，单纯以经济增长速度评定政绩的偏向，更加注重发展质量，更加注重民生保障。要坚持依法施政，健全政府重大决策事项合法性审查制度和行政执法责任制，加强和改进行政复议工作，严格规范行政执法行为。要坚持为民施政，加大政务公开力度，及时回应群众诉求，认真解决好群众反映最多最强烈的切身利益问题。要坚持务实施政，多做经得起历史检验、符合泰安发展实际的实绩，不做应时应景、不利长远发展的虚功。要坚持民主施政，依法向人大报告工作和向政协通报工作，认真落实人大及常委会的决议、决定，自觉接受人大的法律监督和政协的民主监督，认真办理人大代表议案、建议和政协提案。

三要切实改进工作作风。认真落实中央改进工作作风、密切联系群众八项规定和省市委落实办法，健全政府领导干部带头改进作风机制，身体力行，率先垂范。扎实开展党的群众路线教育实践活动，健全改进作风常态化制度，聚焦解决形式主义、官僚主义、享乐主义和奢靡之风，着力解决“慵懒散”和责任落实不到位等突出问题。要健全领导干部深入基层调查研究机制，完善领导干部直接联系和服务群众制度，多到实际困难多、棘手问题多、群众意见大的地方，听民意，问民需，解民忧。要切实改进文风会风，大力精简会议文件。要进一步完善责任落实机制，加大工作督查调度力度，以“钉钉子”的精神抓好政府各项工作的落实。要严格规范公务接待管理，加强审计监督，厉行勤俭节约，反对铺张浪费。要坚持用制度管权管事管人，进一步落实党风廉政建设责任制，健全惩治和预防腐败体系，完善政务和各领域办事公开制度，让每一名政府工作人员切实做到知所循、知所守、知所惧，以廉洁勤政的作风，不断开创泰安各项事业新局面。

各位代表，泰安要实现转型跨越发展，任务非常繁重。让我们紧密团结在以习近平同志为总书记的党中央周围，在中共泰安市委的坚强领导下，不畏艰难，锐意改革，开拓进取，埋头苦干，为加快推进富民强市、建设幸福泰安做出新的更大贡献！
